# Long-term follow-up of congenital melanocytic nevi in a pediatric population: a retrospective study

**DOI:** 10.1590/1984-0462/2025/43/2025160

**Published:** 2025-12-12

**Authors:** Ana Clara Maia Palhano, Débora Natal Moreira Moreira, Chan I Thien, Luciana Paula Samorano, Maria Cecilia Rivitti-Machado, Zilda Najjar Prado de Oliveira

**Affiliations:** a Universidade de São Paulo, SP, Brazil.

**Keywords:** Nevus, pigmented, Nevi and melanomas, Nevus, Nevo pigmentado, Nevos e melanomas, Nevo.

## Abstract

**Objective:**

This study aimed to comprehensively characterize the profile of patients with congenital melanocytic nevi (CMN) through the evaluation of clinical, laboratory, and therapeutic aspects, as well as outcomes related to malignancy. Lesions were classified according to current clinical criteria, considering size, projected adult size, and relevant morphological features

**Methods:**

A retrospective study of patients diagnosed with giant, large, medium, or multiple CMN followed from 1995 to 2022.

**Results:**

Data from 60 patients were evaluated; the mean follow-up period was 5.9 years, and 63.3% were female. According to nevus size, 29.9% were giant, 33.3% large, 28.3% medium, and 8.3% medium multiple. According to location, 36.6% were on the trunk, 30% on extremities, 25% on the head, and 8.3% on other sites. Identified malformations were spina bifida (n=1), failure of fusion of the sacral posterior arch (n=1), and neurocutaneous melanosis (n=1). Melanoma was diagnosed in one patient, and proliferative nodules were found in three cases.

**Conclusions:**

Our findings stress the importance of the long-term follow-up of patients with giant, large, medium, or multiple CMN to detect associated malformations, proliferative nodules, and melanoma.

## RESUMO

**Objetivo:**

Este estudo teve como objetivo caracterizar de forma abrangente o perfil de pacientes com nevo melanocítico congênito, por meio da avaliação de aspectos clínicos, laboratoriais e terapêuticos, além dos desfechos associados à malignidade. As lesões foram classificadas de acordo com critérios clínicos atuais, que consideram o tamanho, a projeção na vida adulta e características morfológicas relevantes.

**Métodos:**

Estudo retrospectivo de pacientes diagnosticados com nevos melanocíticos congênitos gigantes, grandes, médios ou múltiplos, acompanhados entre 1995 e 2022.

**Resultados:**

Foram avaliados dados de 60 pacientes; o tempo médio de seguimento foi de 5,9 anos, e 63,3% eram do sexo feminino. Quanto ao tamanho dos nevos, 29,9% eram gigantes, 33,3% grandes, 28,3% médios e 8,3% médios múltiplos. Com relação à localização, 36,6% estavam no tronco, 30% nos membros, 25% na cabeça e 8,3% em outras regiões. As malformações identificadas foram: espinha bífida (n=1), falha de fusão do arco posterior sacral (n=1), melanose neurocutânea (n=1). Melanoma foi diagnosticado em um paciente e nódulos proliferativos em três casos.

**Conclusões:**

Nossos achados ressaltam a importância do seguimento em longo prazo de pacientes com NMC gigantes, grandes, médios ou múltiplos, visando à detecção de malformações associadas, nódulos proliferativos e melanoma.

## INTRODUCTION

Congenital melanocytic nevi (CMN) are benign intrauterine proliferations of melanocytes derived from the neural crest.^1 2,3,4^ They are present at birth or appear during the first weeks of life,^2^ increasing in thickness over time. NRAS mutations have historically been the most frequently identified genetic alterations in CMN, particularly in large and giant lesions. However, more recent studies have shown that BRAF mutations can also occur in CMN and are not limited to the benign presentations typically associated with acquired nevi. These mutations have been identified in aggressive cases, including large or GCMN with prominent, scattered, or extensive nodularity, as well as in patients with neurocutaneous melanocytosis (NCM). Furthermore, the BRAF V600E mutation has been shown to enhance proliferative activity and influence the histopathological features of CMN in childhood.^5,6^

There are several classification systems for categorizing CMN. More recently, Krengel et al. proposed a CMN categorization system that, in addition to size (diameter) and projected adulthood size (PAS), considers satellite nevus count in the first year of life, anatomic localization, color heterogeneity, surface rugosity, presence of hypertrichosis, and dermal or subcutaneous nodulation (Table 1).^1^

**Table 1.** Classification of congenital melanocytic nevi based on projected adult size, anatomical location, number of satellite nevi, and additional morphologic characteristics.^1^

CMN parameter	Terminology	Definition
CMN projected adult size	“Small CMN”	< 1.5 cm
	“Medium CMN”
	“M1”	1.5–10 cm
	“M2”	>10–20 cm
	“Large CMN”
	“L1”	>20–30 cm
	“L2”	>30–40 cm
	“Giant CMN”
	“G1”	>40–60 cm
	“G2”	>60 cm
	“Multiple medium CMN”	≥3 medium CMN without a single, predominant CMN

**CMN localization**		
CMN of the head	“Face,” “scalp”	
CMN of trunk	“Neck,” “shoulder,” “upper back,” “middle back,” “lower back,” “breast/chest,” “abdomen,” “flank,” “gluteal region,” “genital region”	
CMN of extremities	“Upper arm,” “forearm,” “hand,” “thigh,” “lower leg,” “foot”	
Number of satellite nevi	“S0”	No satellites
	“S1”	20 satellites
	“S2”	20–50 satellites
	“S3”	>50 satellites
Additional morphologic characteristics	“C0,” “C1,” “C2”	None, moderate, marked color heterogeneity
	“R0,” “R1,” “R2”	None, moderate, marked surface rugosity
	“N0,” “N1,” “N2”	None, scattered, extensive dermal, or subcutaneous nodules
	“H0,” “H1,” “H2”	None, notable, marked hypertrichosis (“hairiness”)

CMN: congenital melanocytic nevi.

Histopathologic findings are not specific for CMN, but the presence of nevus cells in the reticular dermis, subcutaneous tissue, or within the epithelium of adnexal structures, perineural tissue, and vessel walls is suggestive of congenital origin.^7^

A major diagnostic challenge lies in distinguishing proliferative nodules (PNs) from melanoma, particularly in large and GCMN. Although PNs are benign lesions that may develop within CMN and clinically resemble melanoma, their histological differentiation can be difficult. Recent reviews have highlighted that classic histologic features of malignancy in adults, such as ulceration, high mitotic activity, and pagetoid spread, may also be present in PNs, without indicating aggressive clinical behavior. Therefore, extreme caution is warranted when diagnosing melanoma in pediatric patients with CMN.^8^

The risk of melanoma in small and medium-sized CMN is considered to be below 1%. In giant CMN (GCMN), the risk is thought to be higher, although two studies have reported rates of 0.7 and 2% for melanoma development during childhood and early adolescence.^9,10^

GCMN may also be associated with extracutaneous malformations such as NCM, Dandy-Walker malformation, and congenital vascular anomalies.^11^ and spina bifida.^12^ A greater risk of NCM in CMN patients has been linked to the presence of more than 20 satellite nevi.^13^ CMN can also carry psychosocial implications, especially in patients with extensive lesions. Surgical excision has traditionally been considered a first-line treatment, although it has not yet been proven to reduce the risk of malignancy.^14^ Other therapeutic approaches, such as dermabrasion, curettage, cryotherapy, electrosurgery, and laser, have also been described. Clinical follow-up is essential, with particular attention to changes in color, thickness, or consistency of the lesion.

The objective of this study was to analyze the profile of patients diagnosed with CMN at the Department of Dermatology of the Hospital das Clínicas of the University of São Paulo Medical School (HC-FMUSP) by assessing clinical, laboratory, and therapeutic aspects, as well as outcomes related to malignancy. A further objective was to categorize lesions in accordance with the cutaneous features described in the classification proposed by Krengel et al.^1^

## METHOD

A retrospective observational study was conducted, including all patients diagnosed with CMN and followed at the Dermatology Department between January 1, 1995, and June 30, 2022, in accordance with the STROBE (Strengthening the Reporting of Observational Studies in Epidemiology) Statement guidelines. Exclusion criteria were incomplete medical records and absence of follow-up information. Data were obtained from medical records, and all patients underwent clinical evaluation and lesion classification. Due to the retrospective nature of the study and its setting in a tertiary referral center, there is a potential selection bias, as more severe or atypical cases may be overrepresented. The clinical parameters analyzed included sex, current age, age at the beginning of dermatologic follow-up, skin phototype, personal and family history of melanoma, family history of CMN, prior neurological investigations, neurological symptoms, presence of NCM, and occurrence of PNs and/or melanoma.

Classification was based on the system proposed by Krengel et al., originally published in 2013. For older cases, clinical classification was performed retrospectively using archived photographs. The classification considered lesion size (diameter), number of satellite nevi during the first year of life (or current number when early data were unavailable), anatomic distribution (back, anterior trunk, genital region, upper limbs, lower limbs, head/neck), heterogeneous pigmentation, surface rugosity, presence of hypertrichosis, and presence of dermal or subcutaneous nodules.

Therapeutic data included clinical observation, partial or complete excision of the CMN, additional interventions such as dermabrasion, laser, or other treatment modalities, and excision of satellite lesions. All excised specimens were submitted to histopathological examination. Malignancy-related outcomes were assessed through the identification of melanomas arising within CMNs. Imaging investigations (magnetic resonance imaging or transfontanellar ultrasound) were performed in patients with axial lesions, multiple CMNs, neurological abnormalities, associated malformations, or a high number of satellite nevi. Only descriptive statistics were applied; no inferential analyses were performed due to the small number of outcomes. This study was approved by the Research Ethics Committee of HC-FMUSP (approval number 3.339.337, CAAE 10041719.0.0000.0068), and written informed consent was obtained from all participants or their legal guardians.

## RESULTS

We identified 84 patients diagnosed with CMN, of whom follow-up data were available for 60 individuals (Table 2). Among these, 38 (63.3%) were female and 22 (36.6%) were male. The mean follow-up duration was 5.9 years. Age at first consultation ranged from the first year of life (n=37, 61%) to 36 years. A family history of large congenital nevus was reported in six patients, including two siblings.

**Table 2.** Summary of clinical and pathological characteristics of patients with congenital melanocytic nevi^1^.

Variable	Category	n (%)
	Female	38 (63.3)
Gender	Male	22 (36.7)
Age at first consultation (years)	Birth	37 (61.7)
	1–5	12 (20.0)
	>5	11 (18.3)
Duration of follow-up	5	25 (41.7)
	5–10	20 (33.3)
	>10	15 (25.0)
Location	Trunk	22 (36.7)
	Extremities	18 (30.0)
	Head/Face	15 (25.0)
	Trunk + genital	5 (8.3)
Classification (Krengel)	Medium (M)	17 (28.3)
	Large (L)	20 (33.3)
	Giant (G)	18 (30.0)
	CNM	5 (8.3)
Satellite nevi (S)	S0	25 (41.7)
	S1–5	11 (18.3)
	S6–30	1 (1.7)
	S31–100	8 (13.3)
	S>100	15 (25.0)
Rugosity (R)	R0 (none)	20 (33.3)
	R1 (mild)	32 (53.3)
	R2 (marked)	8 (13.3)
Hypertrichosis (H)	H0 (none)	8 (13.3)
	H1 (mild)	28 (46.7)
	H2 (marked)	24 (40.0)
Nodules (N)	N0 (none)	40 (66.6)
	N1 (dispersed)	16 (26.7)
	N2 (extensive)	4 (6.7)
Outcomes	Proliferative nodules	3 (5.0)
	Neurocutaneous melanosis	2 (3.3)
	Melanoma	1 (1.6)

CMN: congenital melanocytic nevi.

No malignant transformation was observed in any satellite nevus. Patients with a high number of satellite lesions underwent dermoscopy and total-body photography for monitoring.

**Figure 1.**
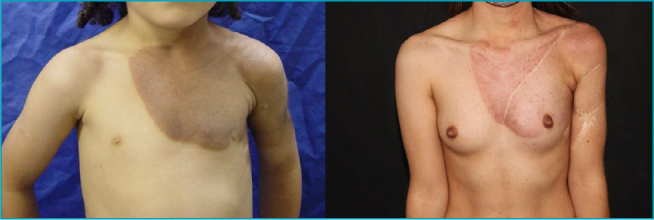
(A) Initial presentation of a 5-year-old patient with an extensive congenital melanocytic nevus involving the left hemithorax and upper arm. Histopathological analysis of an excised nodule suggested a *proliferative nodule with spitzoid features*. (B) Postoperative appearance after eight partial excisions.

Three pediatric patients, two female and one male, aged 5, 7, and 8 years, respectively, developed soft nodules within the lesions. All had giant CMN (G2) located on the trunk. The first patient, a 5-year-old girl, underwent eight partial excisions. Histopathological examination of a nodule on her back initially revealed melanoma in situ. However, upon review, the diagnosis was revised to a PN with spitzoid features arising within the congenital nevus (Figure 1). The second and third patients had histopathological findings consistent with PNs without evidence of malignancy.

**Figure 2.**
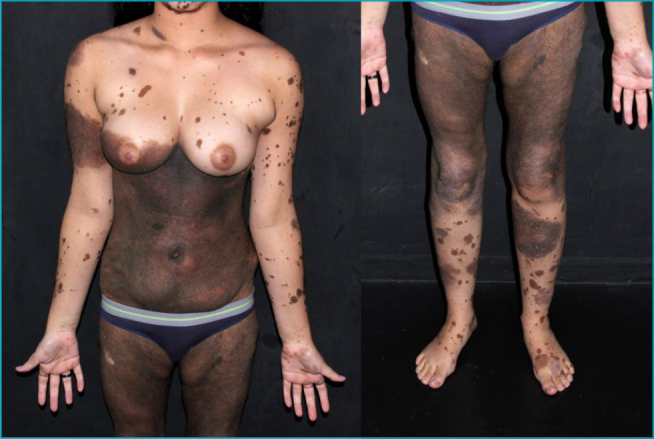
Giant congenital melanocytic nevus involving the head, cervical, and back regions. The patient also presented with spina bifida and neurocutaneous melanosis.

All 16 CMNs located along the midline (2 in the posterior cervical region and 14 in the lumbosacral region) were evaluated with neuraxis magnetic resonance imaging (MRI). Radiologic abnormalities were identified in only one case, corresponding to the previously mentioned 7-year-old female patient with a PN. She presented with an extensive CMN involving the head, cervical, and back regions (Figure 2). Imaging revealed spina bifida at L5 and hypoplasia of the 12th costal arch. The patient also presented with symptomatic epileptic syndrome, with an electroencephalogram showing diffuse disorganization of cerebral electrical activity and epileptiform paroxysms. Brain MRI revealed hyperintensity on T1 and FLAIR sequences in the amygdaloid bodies, consistent with a diagnosis of neurocutaneous melanosis.

During follow-up, one melanoma was identified: a 38-year-old female followed for 11 years. Her GCMN (G2) involved the genital and trunk regions and was classified as C2, R1, N1, and H1, with more than 100 associated satellite nevi (S3). A hard nodule developed on the nevus in the left hip, and histopathology following partial excision revealed melanoma with fusiform cell infiltration into the hypodermis. Immunohistochemistry was positive for S100 and negative for MELAN-A, AML, HHF-35, CD4, AE1/AE3, and HMB-45. The patient died from metastatic melanoma 6 months after diagnosis.

One case of halo nevus was observed in a satellite lesion of a medium-sized CMN (M2); however, histopathological examination following excision showed no evidence of malignancy.

A total of 24 patients (20%) underwent partial excision of their nevi over time. Histopathological evaluation of all excised tissue revealed no malignant transformation.

## DISCUSSION

CMN are rare melanocytic proliferations with variable clinical behavior that warrant long-term, multidisciplinary follow-up. From a molecular standpoint, the majority of large and GCMN harbor postzygotic somatic mutations in the NRAS gene, particularly involving codon 61, which has been detected in approximately 80% of cases.^15,16^ In addition to NRAS, recent findings have highlighted that BRAF mutations can also be present in congenital lesions, including some with aggressive features such as extensive nodularity and proliferative activity.^5,6^ This heterogeneity reinforces the complexity of CMN and underscores the importance of integrating genetic, clinical, and histopathological data for accurate risk stratification and individualized patient care, and the development of reliable predictive tools — whether phenotypic or genotypic — for identifying patients at higher risk for complications such as NCM or melanoma remains an ongoing challenge in CMN research.

There is no consensus in the literature regarding sex predominance among affected individuals, although in our series, there was a higher frequency of female patients. Our study did not include small CMN, which are more frequent,^14^ as our institution is a tertiary referral center. Among the patients evaluated, 28.3% were classified as having medium-sized CMN, 33.3% as large, and 29.9% as giant.

Regarding nevus location, 22 were found on the trunk, followed by 18 on the extremities and 15 on the head. This distribution is consistent with previous studies reporting a predominance of lesions on the trunk and abdomen, with a lower frequency in the head and neck regions.^17^

Neurocutaneous melanosis is a rare congenital disorder that may be either asymptomatic or symptomatic, potentially progressing to neuropsychomotor delay, seizures, and growth restriction. It results from an excessive accumulation of melanocytes and melanin in the leptomeninges. Risk factors for its development include large or GCMN located on the head, neck, or posterior midline, and the presence of more than 20 satellite nevi.^18,19^ Diagnosis is made by MRI of the neuraxis, with the highest diagnostic sensitivity occurring between 4 and 6 weeks of life. However, the risk-benefit ratio of performing the exam at this age remains debatable and controversial. Additionally, many patients begin follow-up after this

optimal diagnostic window, as observed in our study, with some individuals starting follow-up only after the first year of life. This may partially explain the low rate of neurocutaneous melanosis observed in our sample.

Melanoma is the most serious complication of CMN due to its high morbidity and mortality. Nevertheless, it remains relatively rare, as demonstrated in our series, where 1 out of 60 patients (1.6%) developed melanoma and died from the disease. Among patients with large and giant nevi (n=32), the melanoma incidence was 3.1%. The average age for melanoma development in CMN, according to the literature, is approximately 7 years. This supports the notion that large and GCMN carry a higher risk of malignancy during childhood, while medium-sized nevi have an increased risk during adolescence. In contrast, the risk associated with small CMN is generally higher in adulthood, particularly after 60 years of age.^6^ These findings underscore that small and GCMN differ in their malignancy risk profiles, and the need for close monitoring of small lesions remains a controversial issue. Some studies have reported malignancy in small nevi.^20^ In our series, the melanoma occurred in a patient with a GCMN who developed melanoma at age 38. These findings emphasize the importance of lifelong surveillance; however, it is important to note that the low number of events (1 melanoma, 2 NCM cases) limits statistical power and the generalizability of the findings. Moreover, the follow-up period in this study may have been insufficient to capture all cases of malignant transformation, with a mean follow-up of 5.9 years (ranging from 10 months to 27 years).

As for malformations, spina bifida with incomplete fusion of the posterior sacral arch was identified in one patient with a midline CMN, underscoring the importance of screening for underlying spinal anomalies when lesions are located along the axial midline.

This study presents inherent limitations that should be acknowledged. The retrospective and single-center design may have introduced selection bias, especially given the setting in a tertiary referral hospital. The relatively low incidence of key outcomes, such as melanoma and NCM, limited the statistical power to identify independent risk factors. Follow-up duration was also variable and may have been insufficient in some cases to detect late-onset complications. However, despite these limitations, this study provides valuable insights into the long-term clinical outcomes of patients with CMN over a 27-year period, offering one of the largest single-center experiences from Latin America. The detailed phenotypic characterization and use of a standardized classification system strengthen the clinical relevance of the findings and may inform future research and patient care strategies.

In conclusion, CMN are heterogeneous lesions that require lifelong and multidisciplinary follow-up, especially in patients with large and giant forms due to the increased risk of PNs,

NCM, and melanoma. In our study, malignant transformation was uncommon, yet its occurrence reinforces the need for vigilant surveillance. The use of a standardized classification system enabled precise phenotypic characterization, supporting more accurate risk assessment.

## Data Availability

The database that originated the article is available with the corresponding author.

## References

[1] Krengel S, Scope A, Dusza SW, Vonthein R, Marghoob AA (2013). New recommendations for the categorization of cutaneous features of congenital melanocytic nevi. J Am Acad Dermatol.

[2] Krengel S (2005). Nevogenesis--new thoughts regarding a classical problem. Am J Dermatopathol.

[3] Alikhan A, Ibrahimi OA, Eisen DB (2012). Congenital melanocytic nevi: where are we now? Part I. Clinical presentation, epidemiology, pathogenesis, histology, malignant transformation, and neurocutaneous melanosis.. J Am Acad Dermatol.

[4] Ruiz-Maldonado R (2004). Measuring congenital melanocytic nevi. Pediatr Dermatol.

[5] Chen J, Zhang G, Liu X, Tu P (2023). The association of BRAF V600E gene mutation with proliferative activity and histopathological characteristics of congenital melanocytic nevi in children. An Bras Dermatol.

[6] Salgado CM, Basu D, Nikiforova M, Bauer BS, Johnson D, Rundell V (2015). BRAF mutations are also associated with neurocutaneous melanocytosis and large/giant congenital melanocytic nevi. Pediatr Dev Pathol.

[7] Mark GJ, Mihm MC, Liteplo MG, Reed RJ, Clark WH (1973). Congenital melanocytic nevi of the small and garment type. Clinical, histologic, and ultrastructural studies. Hum Pathol.

[8] Salgado CM, Tomás-Velázquez A, Reyes-Mÿgica M (2025). Congenital melanocytic neoplasms: clinical, histopathological and recent molecular developments. Virchows Arch.

[9] Kinsler VA, Birley J, Atherton DJ (2009). Great ormond street hospital for children registry for congenital melanocytic naevi: prospective study 1988–2007. Part 1 – Epidemiology, phenotype and outcomes. Br J Dermatol.

[10] Krengel S, Hauschild A, Schfer T (2006). Melanoma risk in congenital melanocytic naevi: a systematic review. Br J Dermatol.

[11] Wu PA, Mancini AJ, Marghoob AA, Frieden IJ (2008). Simultaneous occurrence of infantile hemangioma and congenital melanocytic nevus: coincidence or real association?. J Am Acad Dermatol.

[12] Foster RD, Williams ML, Barkovich AJ, Hoffman WY, Mathes SJ, Frieden IJ (2001). Giant congenital melanocytic nevi: the significance of neurocutaneous melanosis in neurologically asymptomatic children. Plast Reconstr Surg.

[13] Marghoob AA, Dusza S, Oliveira S, Halpern AC (2004). Number of satellite nevi as a correlate for neurocutaneous melanocytosis in patients with large congenital melanocytic nevi. Arch Dermatol.

[14] Ibrahimi OA, Alikhan A, Eisen DB (2012). Congenital melanocytic nevi: where are we now? Part II. Treatment options and approach to treatment. J Am Acad Dermatol.

[15] Kinsler VA, Krengel S, Riviere JB, Waelchli R, Chapusot C, Al-Olabi L (2014). Next-generation sequencing of nevus spilus type congenital melanocytic nevus: exquisite genotype-phenotype correlation in mosaic RASopathies. J Invest Dermatol.

[16] Kinsler VA, Thomas AC, Ishida M, Bulstrode NW, Loughlin S, Hing S (2013). Multiple congenital melanocytic nevi and neurocutaneous melanosis are caused by postzygotic mutations in codon 61 of NRAS. J Invest Dermatol.

[17] Warner PM, Yakuboff KP, Kagan RJ, Boyce S, Warden GD (2008). An 18-year experience in the management of congenital nevo-melanocytic nevi. Ann Plast Surg.

[18] Recio A, Felix V, Campos Y (2017). Congenital melanocytic nevus syndrome: a case series. Actas Dermosifiliogr.

[19] Marchesi A, Leone F, Sala L, Gazzola R, Vaienti L (2012). Giant congenital melanocytic naevi: a review of the literature. Pediatr Med Chir.

[20] Tannous ZS, Mihm MC, Souber AJ, Duncan LM (2005). Congenital melanocytic nevi: clinical and histopathologic features, risk of melanoma, and clinical management. J Am Acad Dermatol.

